# Korean Version of a Model to Estimate Survival in Ambulatory Patients with Hepatocellular Carcinoma (K-MESIAH)

**DOI:** 10.1371/journal.pone.0138374

**Published:** 2015-10-21

**Authors:** Byung-Ho Nam, Joong-Won Park, Sook-Hyang Jeong, Sang Soo Lee, Ami Yu, Bo Hyun Kim, W. Ray Kim

**Affiliations:** 1 Department of Cancer Control and Policy, Graduate School of Cancer Science and Policy, National Cancer Center, Goyang, Republic of Korea; 2 Center for Liver Cancer, National Cancer Center, Goyang, Republic of Korea; 3 Department of Internal Medicine, Seoul National University Bundang Hospital, Seoul National University, College of Medicine, Seongnam, Republic of Korea; 4 Biometric Research Branch, National Cancer Center, Goyang, Republic of Korea; 5 Division of Gastroenterology and Hepatology Stanford University, Stanford, California, United States of America; Kaohsiung Chang Gung Memorial Hospital, TAIWAN

## Abstract

**Background and Aim:**

A model to estimate survival in ambulatory hepatocellular carcinoma patients (MESIAH) is useful for estimating patient prognosis but needs improvement for Korean patients, most of whom have a hepatitis B virus. We aimed to modify the MESIAH for better prognostication through enhancing calibration for Korean patient population (K-MESIAH).

**Methods:**

Utilizing a cohort of 1,969 hepatocellular carcinoma (HCC) patients from the National Cancer Center of Korea between 2004 and 2009, a survival prediction model was developed using the Cox proportional hazards model. The model’s performance was evaluated using C-statistical and χ^2^-statistical analyses. External validation was performed using an independent cohort of 328 patients from the Seoul National University Bundang Hospital.

**Results:**

To develop the K-MESIAH, etiology was added to the original risk factors (age, Model for Endstage Liver Disease, albumin, size of the largest nodule, number of tumor nodules, vascular invasion, metastasis, and alpha fetoprotein) in the MESIAH. From the internal validation study, the C-statistics and χ^2^-statistics for one-, three-, and five-years of survival were 0.83 (95% Confidence Interval: 0.82−0.85), 49.07; 0.81 (95% Confidence Interval: 0.79−0.82), 28.95; and 0.80 (95% Confidence Interval: 0.79−0.81), 20.93, respectively. The K-MESIAH also showed a high prediction ability for the external validation cohort.

**Conclusions:**

A survival prediction model for Korean HCC patients was developed and validated to have a high level of performance. This K-MESIAH may be more useful in clinical practice and personalized care in a hepatitis B virus endemic area.

## Introduction

An accurate staging system is necessary to predict the prognosis of patients with cancer and to guide the therapeutic approach.[1] Although the tumor, node, and metastasis (TNM) system represents the extent and severity of the tumor and is the most widely used cancer staging system, patients with hepatocellular carcinoma (HCC) need to be assessed for both liver function and tumor extent.[2] For the majority of HCC patients, underlying liver cirrhosis or chronic hepatitis impacts prognosis in addition to the extent and severity of their tumor.[3] The Barcelona Clinic Liver Cancer (BCLC) staging system takes into account liver function using scoring systems like the Child-Pugh (CP) classification in addition to assessing performance status, cancer-related symptoms, and the tumor extent.[4] The BCLC staging has become a leading staging system by which HCC patients are stratified and recommended for therapeutics.[5]

Recently, a model to estimate survival in ambulatory HCC patients (MESIAH)[6] was proposed based on two cohorts, one from the Mayo Clinic in Rochester, United States (derivation cohort) and the other from the National Cancer Center, Korea (validation cohort).[7] This MESIAH uses the Model for End-Stage Liver Disease (MELD) score[8] to gauge liver dysfunction and includes objective and reproducible clinical parameters and tumor characteristics. Compared with other scoring systems, the MESIAH provides a wide range of scores to allow stratification of HCC patients with significantly different prognoses to provide survival probabilities, which is useful in clinical practice.[6]

The calibration of the MESIAH score needs to be improved for use in Korean HCC patients, most of whom have hepatitis B virus (HBV)-related chronic liver disease. In this study, we modified the MESIAH for better prognostication of survival specifically for Korean HCC patients in an HBV-endemic area and validated the modified system, which we termed the K-MESIAH.

## Methods

### Study population and data collection

This study was approved by the Institutional Review Board (IRB) of the Seoul National University Bundang Hospital (SNUBH), Seongnam, South Korea and the IRB of the National Cancer Center, Goyang, South Korea. The written informed consent was waived and the patient records/information was anonymized and de-identified prior to analysis. To derive the K-MESIAH, a cohort of 1,969 newly diagnosed HCC patients without prior anti-tumor therapy and treated at the Center for Liver Cancer, National Cancer Center (NCC, Goyang, South Korea) between January 2004 and December 2009[9] was used (termed the development cohort). To validate the K-MESIAH after development, 328 newly diagnosed HCC patients without any prior anti-cancer therapy and treated at the SNUBH from May 2003 to April 2010 were used. All patients were followed until March 2012. For the development patient cohort, the medical records were prospectively collected, and the clinical and tumor characteristics were retrospectively reviewed. For the validation cohort, the clinical and radiological data were obtained from electronic medical records and radiological images, and retrospectively analyzed. A diagnosis of HCC was made based on histology and/or clinico-radiologic evidence according to the practice guidelines of the Korea Liver Cancer Study Group-NCC, Korea.[10] The Modified Union for International Cancer Control (mUICC) stages were used for tumor staging,[11] and the BCLC staging system was used for clinical staging.[4] The CP and MELD scoring systems[8] were used for clinical diagnosis and classification of the severity of liver dysfunction, respectively.

The MELD score was calculated according to the original formula without rounding and without setting lower and upper bounds for the variables or final score. The MELD scores <13 were set to 13. The numbers of nodules greater than or equal to 5 were recorded as 5. The size of the largest nodule was recorded as follows: 1 = ≤1 cm, 2 = >1−2 cm, 3 = >2−3 cm, 4 = >3−5 cm, 5 = >5−10 cm, 6 = >10−15 cm, 7 = >15−20 cm, and 8 = >20 cm. The serum alpha fetoprotein (AFP) level was capped at 10,000 ng/mL.

### Development of survival prediction model

The Cox proportional hazard model was employed to develop a multivariate model to predict one-, three-, and five-year survival rates for Korean HCC patients. The overall survival time is defined as the time between the first diagnosis date and the death. Crude and age-adjusted analyses were performed to identify potential risk factors. Three selection procedures (forward, backward, and stepwise) were used to select the best fit model. A statistical significance level of 0.10 was used to select variables into the model. A hierarchical variable selection method was used to compare models with different sets of variables. Parsimonious and best performance principles were applied to select the final model.

The variables considered during the model development stage[12] included age, sex, Eastern Cooperative Oncology Group (ECOG) performance score, etiology, the size of the largest nodule, CP Score (CPS), albumin, portal vein tumor thrombosis (vascular invasion), creatinine, the number of nodules, bilirubin, international normalized ratio (INR), AFP, extra hepatic metastasis (EHS), and the MELD score.

### Validation of survival prediction model

Both internal and external validations were performed to evaluate the model’s discrimination and calibration abilities. Discrimination is defined as a model’s ability to correctly distinguish non-events and events. This can be quantified by calculating the C-statistic developed for the survival model.[13] The C-statistic is a concordance measure analogous to the receiver operating characteristic (ROC) curve, which indicates the probability that a model produces higher risks for those who develop events compared with those who do not develop events.[14] All statistical analyses were performed using SAS, version 9.2 (SAS institute, Cary, NC). A SAS macro was used to calculate the C-statistic with 95% confidence intervals (CIs).

Calibration measures how closely the predicted probabilities agree numerically with the actual outcomes. A Hosmer–Lemeshow (H-L) type χ^2^-statistic was used.[13] This χ^2^-statistic was calculated by first dividing the data into 5 groups (quintiles; the lowest, medium-low, medium, medium-high, and the highest risk group) by the predicted probabilities produced by the model in ascending order. Then, for each quintile, the average predicted probabilities were compared to the actual event rate estimated using the Kaplan–Meier approach.[15] Graphs were generated using the Stata statistical software, version 12 (STATA, College Station, TX).

Other performance measures such as Likelihood Ratio (LR) and the Akaike information criterion (AIC) were used to compare the K-MESIAH and the MESIAH

## Results

### Model development cohort

#### Baseline Characteristics

The baseline characteristics of the development cohort are summarized in Table 1. The cohort was predominantly male and the median age was 56 (interquartile range [IQR] = 49−64) years. HBV was the major etiology (74.6%) and the median MELD score was 9.27 (IQR = 7.86−11.11). Approximately half of the patients had a single nodule and the tumor sizes varied. More than one third of the patients had vascular invasion. Most of the patients had good ECOG performance (0–1) and did not have extrahepatic metastasis. The median survival time was 21.4 months and 1,333 patients (67.70%) were expired.

**Table 1:** Baseline characteristics and univariate analysis in the development cohort (n = 1,969).

Risk Factor	Median [IQR] or n (%)	Hazard Ratio (95% CI)
Age (in decades)	5.6 [4.9–6.4]	0.969 (0.919, 1.021)
Sex		
Male	1639 (83.24)	1.316 (1.131, 1.531)
Female	330 (16.76)	
Etiology		
HBV	1469 (74.61)	
HCV	184 (9.34)	0.847 (0.702, 1.022)
Alcohol	144 (7.31)	0.737 (0.594, 0.915)
NBNCNA^†^	172 (8.74)	0.891 (0.732, 1.084)
Bilirubin	0.9 [0.6–1.3]	1.045 (1.032, 1.058)
INR	1.15 [1.06–1.27]	1.062 (0.985, 1.144)
Creatinine	1.0 [0.9–1.1]	0.919 (0.756, 1.117)
Albumin	3.8 [3.4–4.2]	0.512 (0.470, 0.557)
MELD	9.27 [7.86–11.11]	1.044 (1.022, 1.067)‡
CPS	5 [5–6]	1.268 (1.221, 1.317)
ECOG		
0	853 (43.32)	
1	1052 (53.43)	2.474 (2.200, 2.782)
2	61 (3.10)	3.969 (3.000, 5.251)
3	3 (0.15)	47.919 (15.243, 150.643)
Number of nodules		
1	1008 (51.19)	
2	312 (15.85)	1.178 (1.003, 1.383)
3	136 (6.91)	1.581 (1.285, 1.945)
4	72 (3.66)	1.755 (1.339, 2.301)
≥ 5	441 (22.40)	3.004 (2.637, 3.421)
Size of the largest nodule		
≤ 1 cm	49 (2.49)	
>1−2 cm	250 (12.70)	1.112 (0.663, 1.866)
>2−3 cm	293 (14.88)	1.523 (0.920, 2.522)
>3−5 cm	387 (19.65)	2.211 (1.351, 3.619)
>5−10 cm	530 (26.92)	4.451 (2.740, 7.231)
>10−15 cm	310 (15.74)	8.013 (4.905, 13.091)
>15−20 cm	126 (6.40)	12.393 (7.442, 20.636)
> 20 cm	24 (1.22)	13.240 (7.060, 24.830)
AFP (ng/mL)	171.6 [14.0–3272.0]	1.204 (1.180, 1.229)§
Vascular invasion		
none	1296 (65.82)	
positive	673 (34.18)	5.313 (4.739, 5.957)
Extrahepatic metastasis		
none	1611 (81.82)	
positive	358 (18.18)	3.749 (3.303, 4.256)
HCC type		
well-defined	1329 (67.50)	
ill-defined	640 (32.50)	4.021 (3.594, 4.500)

^†^non-HBV, non-HCV and non-alcoholic.

^‡^MELD scores: < 13 set to 13.

^§^ln(AFP) with AFP capped at 10,000 ng/mL.

IQR, Interquartile range; CI, Confidence interval; HBV, Hepatitis B virus; HCV, Hepatitis C virus; INR, International normalized ratio; MELD, Model for end-stage liver disease; CPS, Child-Pugh score; ECOG, Eastern Cooperative Oncology Group; AFP, Alpha fetoprotein; HCC, Hepatocellular carcinoma.

#### Risk Prediction Model (K-MESIAH)

Univariate and multivariate analyses were performed using the Cox regression model (Table 2). In the univariate analyses, directions of the hazard ratios for age, sex, CPS, MELD, bilirubin, albumin, tumor number, tumor size, AFP, extrahepatic metastasis, and vascular invasion were in agreement with the results of the MESIAH, though their magnitudes were slightly different. INR and creatinine did not show significant association with survival from the univariate analysis.

**Table 2:** Risk prediction model: K-MESIAH.

Risk Factor	K-MESIAH		
	*β*	Hazard Ratio (95% CI)	P -value
Age (in decades)	0.089	1.093 (1.030, 1.161)	<0.01
MELD^†^	0.025	1.025 (0.998, 1.053)	0.07
Albumin	-0.558	0.573 (0.521, 0.630)	<0.01
Number^‡^	0.114	1.121 (1.085, 1.158)	<0.01
Size^§^	0.274	1.315 (1.256, 1.376)	<0.01
Vascular invasion	0.992	2.697 (2.360, 3.081)	<0.01
Metastasis	0.468	1.596 (1.390, 1.833)	<0.01
AFP^¶^	0.078	1.081 (1.057, 1.105)	<0.01
Etiology			
HCV	-0.135	0.874 (0.716, 1.066)	0.184
Alcoholic	-0.202	0.817 (0.655, 1.021)	0.075
NBNCNA	-0.113	0.894 (0.730, 1.094)	0.275

^†^MELD scores: < 13 set to 13.

^‡^Number of nodules: 1 = 1 / 2 = 2 / 3 = 3 / 4 = 4 / 5 = ≥ 5.

^§^Size of the largest nodule: 1 = ≤ 1cm / 2 = >1−2 / 3 = >2−3 / 4 = >3−5 / 5 = >5−10 / 6 = >10−15 / 7 = >15−20 / 8 = > 20.

^¶^ln(AFP) with AFP capped at 10,000 ng/mL.

K-MESIAH, Korean model to estimate survival in ambulatory hepatocellular carcinoma patients; CI, Confidence interval; MELD, Model for end-stage liver disease; AFP, Alpha fetoprotein; HCV, Hepatitis C virus; NBNCNA, Non-HBV, non-HCV and non-alcoholic.

$\begin{array}{l} \text{K-MESIAH SCORE}=0.089\times \left(\text{Age in Decades}\right)+0.025\times \left(\text{MELD}\right)-0.558\times \left(\text{Albumin}\right)\\ \;+0.114\times \left(\text{Tumor Number}\right)+0.274\times \left(\text{Tumor Size}\right)+0.992\times \left(\text{Vascular Invasion}\right)\\ \;+0.468\times \left(\text{Metastasis}\right)+0.078\times \left(\text{AFP}\right)-0.135\times \left(\text{HCV}\right)\\ \;-0.202\times \left(\text{Alcoholic}\right)-0.113\times \left(\text{NBNCNA}\right) \end{array}$

In the multivariate analysis, the original combination of risk factors in the MESIAH showed as good as or better than other combinations of risk factors selected by forward, backward, or stepwise variable selection methods. We determined to use the risk factors in the MESIAH as the predictors in the final model based on the principle of parsimony, and added etiology, which improves the model performance compared to HCC type for better calibration in Korean HCC patients.

The final K-MESIAH model includes age, the MELD scores, albumin, the number of nodules, the size of the largest nodule, vascular invasion, metastasis, AFP, and etiology. Application of the risk score in individual patients allows calculation of expected survival. For a hypothetical patient, *S*_0_(*t*) gives the estimated survival probabilities with a given risk score, which is the median risk score of the patients in the development cohort (Table 3). We calculated the risk probability for the development cohort using the K-MESIAH as follows:

$$P\left(t\right)=1-S_{0}{\left(t\right)}^{\text{exp}\left(\text{K-MESIAH score}-0.99\right)}$$

where the estimates of the baseline survival function *S*_0_(*t*) were 0.664, 0.347 and 0.220 at one-, three-, and five-years, and 0.99 is the average risk score.

**Table 3:** Expected survival probability according to the K-MESIAH in a hypothetical patient.

Risk Factor	Value	P(t)(%)^†^		
		1-year	3-year	5-year
Age	45	46.0	79.6	89.8
Sex	Male			
Etiology	HBsAg positive			
MELD score	9			
INR	1.2			
Total Bilirubin	1.0 mg/dL			
Creatinine	1.2mg/dL			
Albumin	3.6 mg/dL			
AFP	1693 ng/mL			
Tumor size	8 cm			
Tumor number	1			
Vascular invasion	positive			
Metastasis	none			

HBsAg, Hepatitis B serum antigen; MELD, Model for end-stage liver disease; INR, International normalized ratio; AFP, Alpha fetoprotein; P(t), risk probability.

^†*P*(*t*) = 1−*S*_0_(*t*)^exp(K-MESIAH score−0.99)^^

### Internal validation

**Fig 1:**
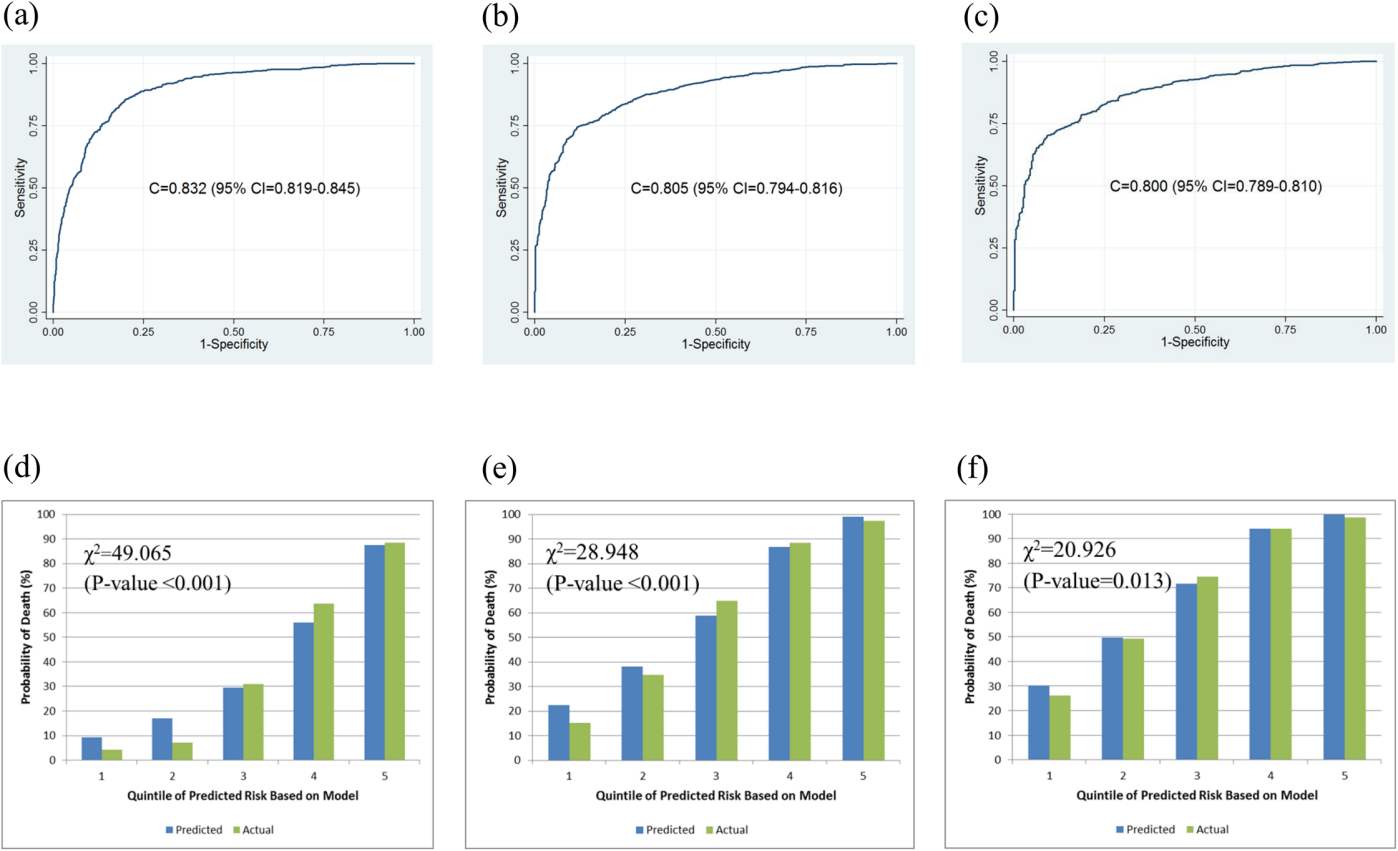
Discrimination and Calibration Ability of the K-MESIAH in the internal validation cohort. Discrimination ability in the internal validation cohort at one-(a), three-(b), and five-(c) years after diagnosis with hepatocellular carcinoma, and calibration bar plot (how closely the predicted probabilities agree numerically with the actual outcomes) of the K-MESIAH in the development cohort at one-(d), three-(e), and five-(f) years. Groups were divided by quintiles of risk probability (1 = lowest / 2 = med-low / 3 = medium / 4 = med-high / 5 = highest).

The C-statistics of the K-MESIAH for one, three, and five-years were 0.832 (95% CI; 0.819−0.845), 0.805 (95% CI; 0.794−0.816), and 0.800 (95% CI; 0.789−0.810), and the H-L type χ^2^-statistics were 49.065 (P-value < 0.001), 28.948 (P-value < 0.001), 20.926 (P-value = 0.013) (Fig 1). The K-MESIAH showed good discrimination ability for the development cohort. The LR and AIC for the K-MESIAH were 1357.9 and 17448.7. For MESIAH, the LR and AIC were 1263.0 and 17523.5.

### External validation

The characteristics of the validation cohort were summarized at Table 4. The median AFP was lower than in the development cohort. Fewer patients had vascular invasion and metastasis. The categorization of tumor number and size in the validation cohort was slightly different from those of the MESIAH. We assigned low values to them based on category intervals. The MELD score and AFP were transformed in the same way as in the MESIAH. With the median survival time of 55.0 months, the mortality rate was 35.67% (117 deaths).

**Table 4:** External validation cohort (SNUBH patients n = 328).

K-MESIAH Variable	Median [IQR] or n (%)
Age (in decades)	6.1 [5.3–6.8]
MELD	7.87 [5.72–10.36]
Albumin	3.8 [3.3–4.1]
Number of nodules^†^	
1	164 (50.00)
2**−**3	73 (22.26)
≥ 4	91 (27.74)
Size of the largest nodule^‡^	
< 2 cm	80 (24.39)
2**−**5 cm	119 (36.28)
> 5 cm	129 (39.33)
Vascular invasion	
No	249 (75.91)
Yes	79 (24.09)
Metastasis	
No	311 (94.82)
Yes	17 (5.18)
AFP (ng/mL)	42.7 [8.0–558.0]
Etiology	
HBV	227 (69.21)
HCV	45 (13.72)
Alcoholic	38 (11.59)
NBNCNA	18 (5.49)

^†^Number of nodules: 1 = 1 / 2 = 2−3 / 4 = ≥ 4.

^‡^Size of the largest nodule: 1 = < 2cm / 3 = 2−5 / 5 = > 5.

SNUBH, Seoul National University Bundang Hospital; K-MESIAH, Korean model to estimate survival in ambulatory hepatocellular carcinoma patients; IQR, Interquartile range; MELD, Model for end-stage liver disease; AFP, Alpha fetoprotein; HBV, Hepatitis B virus; HCV, Hepatitis C virus; NBNCNA, Non-HBV, non-HCV and non-alcoholic

**Fig 2:**
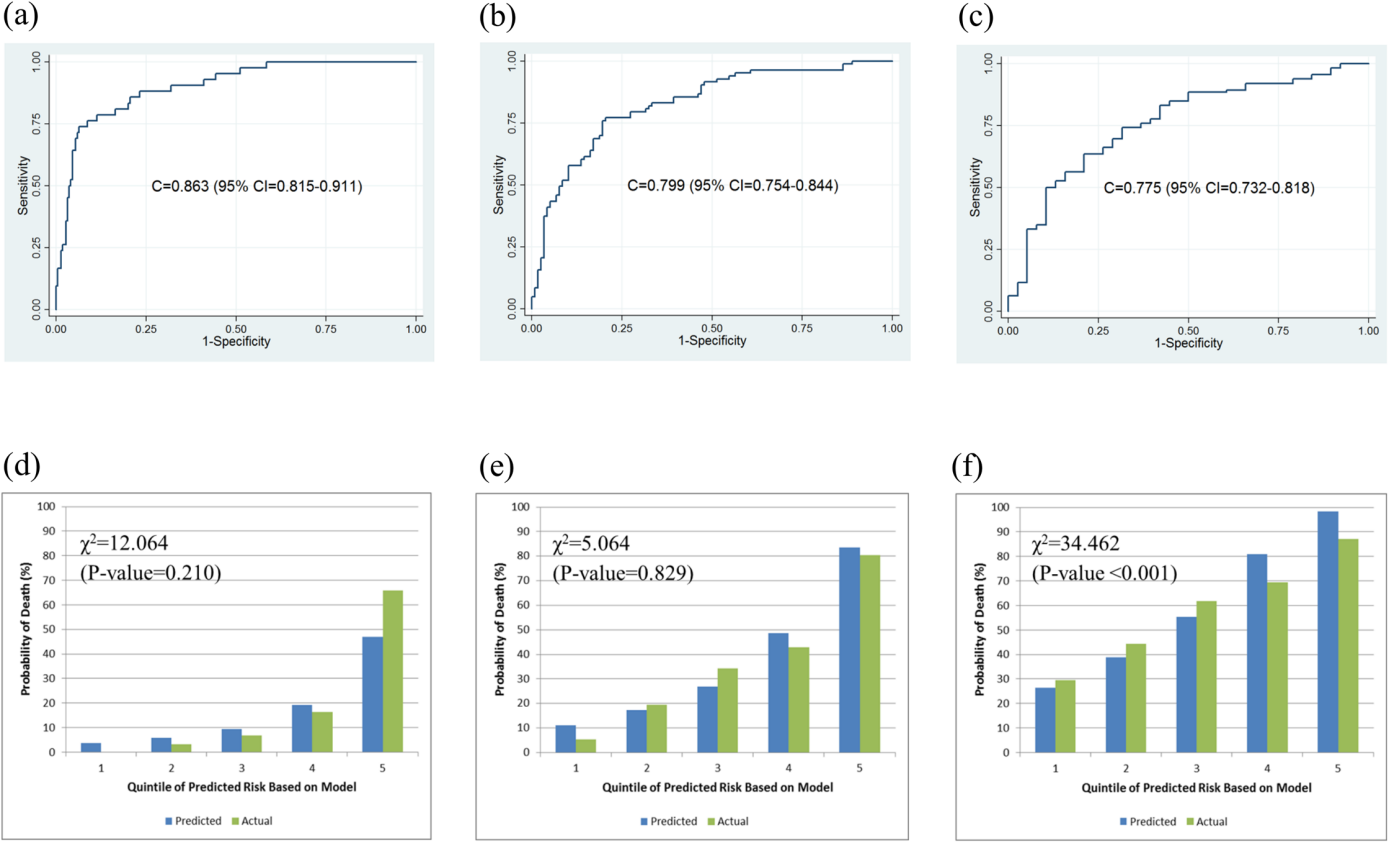
Discrimination and Calibration ability of the K-MESIAH in the external validation cohort. Discrimination ability at one-(a), three-(b), and five-(c) years after diagnosis with hepatocellular carcinoma, and calibration bar plot at one-(d), three-(e), and five-(f) years. Groups were divided by quintiles of risk probability (1 = lowest / 2 = med-low / 3 = medium / 4 = med-high / 5 = highest).

The K-MESIAH showed high performance with a good discrimination ability in the validation cohort. The C-statistic (95% CI) at one year was 0.863 (0.815−0.911), which surpassed that of the development cohort. The calibration ability of the K-MESIAH for the validation cohort at one- and three-years was excellent (χ^2^ = 12.064 [P-value = 0.210] and χ^2^ = 5.064 [P-value = 0.829], respectively (Fig 2).

### Cumulative incidence rates for five risk groups

**Fig 3:**
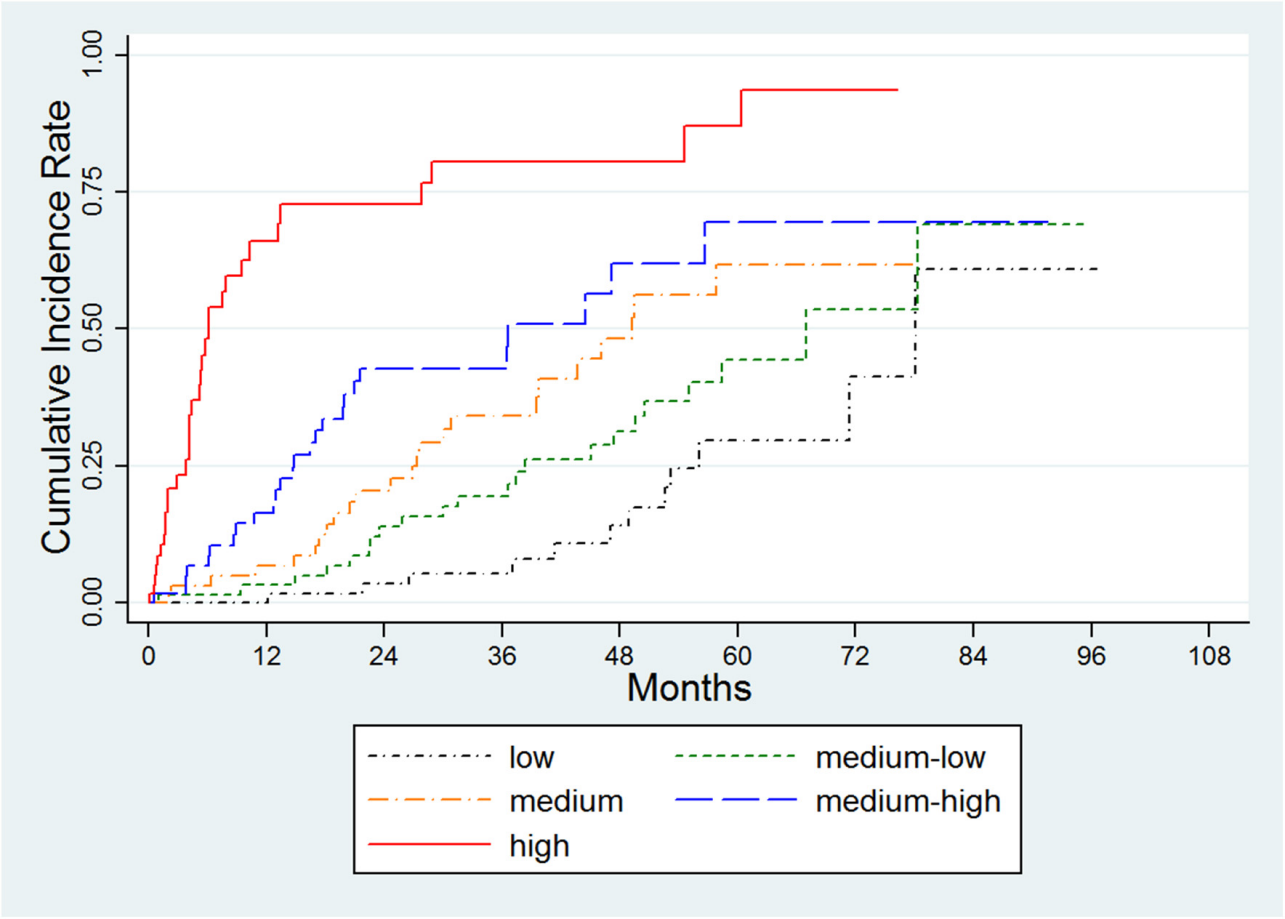
Cumulative incidence rates of death for the five risk groups in the development cohort. Groups were divided by quintiles of risk probability, in which the med-low / med / med-high / and highest risk groups had 2.17 (95% Confidence Interval [CI]: 1.70–2.78) / 4.83 (95% CI: 3.84–6.08) / 11.27 (95% CI: 8.99–14.14) / and 26.56 (95% CI: 21.07–33.48) -fold higher incidence of hepatocellular carcinoma compared to the lowest risk group, respectively.

Fig 3 shows the cumulative incidence rates of death in the development cohort for groups divided by quintiles of the risk probability in an ascending order. At 5 years, the cumulative incidence rate of the various risk groups was 26.14%, 49.34%, 74.47%, 93.92%, and 98.52%, respectively. The medium-low, medium, medium-high, and highest risk group had significantly higher hazards compared to the lowest risk group (hazard ratios were 2.17, 4.83, 11.27, and 26.56 respectively).

## Discussion

Recently, we reported the first validation study of the MESIAH scoring system to predict survival in a cohort of HCC patients[16] and concluded that the MESIAH accurately estimated the overall survival of Korean HCC patients. The MESIAH scoring system showed a higher degree of discrimination and better likelihood ratios (LR), χ^2^, and Akaike information criterion (AIC) compared to the BCLC staging system.[16] However, during this validation study, we found that the MESIAH required improved calibration for Korean HCC patients with HBV.

Therefore, in this study, we developed a model with better calibration for HCC population with dominant HBV etiology while maintaining at least the same risk factors included in the original MESIAH. In our final K-MESIAH, etiology was added to the original MESIAH risk factors, which improves the model performance for Korean HCC patients. Tumor type was one candidate risk factor to be added, but was excluded from this model based on the principle of parsimony. The cohort for developing the K-MESIAH is the same cohort used to validate the MESIAH.[16] The C-statistics of the K-MESIAH (Fig 1) are better than those of the MESIAH (overall 0.792 [95% CI, 0.782–0.803]).[16] The K-MESIAH showed reasonably good calibration ability in overall even though a slight over-prediction was observed for lower risk groups at the one year time point. With respect to the external validation, the K-MESIAH showed good performance in both discrimination and calibration ability (Fig 2). A slight over-prediction was observed for five year time point in higher risk group probably due to more high risk patients in the development cohort compared to the validation cohort (i.e. higher MELD score, larger tumors, higher AFP level, more vascular invasion, and more metastasis) and relatively shorter follow-up in the validation cohort.

The MESIAH was developed based on the derivation cohort of the Mayo Clinic in Rochester, United States (1994–2008) and the validation cohort of the National Cancer Center, Korea (2000–2003). In the MESIAH derivation cohort, there were two main etiologies; HBV (84 patients) and HCV (384 patients). The hazard ratio of HBV (0.906) was almost the same as that of HCV (1.028) for survival and the etiology was not statistically significant in the multivariate analysis. Therefore, etiology was excluded from the MESIAH scoring system. In contrast to the MESIAH, there were four main etiologies in the development cohort of the K-MESIAH; HBV (1469 patients), HCV (184 patients), alcohol (144 patients), and non-HBV non-HCV non-alcohol (172 patients) (Table 1). Etiology showed a marginally significant P-value (0.0614) in the multivariate analysis for survival.[9] In the K-MESIAH development, taking etiology into account improves the model performance. The K-MESIAH showed better performance in terms of LR and AKI compared to the MESIAH by adding etiology. In Asia, where there is a high prevalence of HBV infection, HCC patients show different clinical characteristics compared to those in Western countries.[17,18] HBV antiviral therapy in HBV-associated HCC patients reportedly prolongs survival[19] and our recent cohort showed a significant improvement in survival when treated with antiviral therapy. Whether HBV- and HCV-related HCCs have a different prognosis remains controversial, but HBV-related HCC in an Italian cohort was more aggressive than HCV-related tumors[20] and our recent HCC cohort also showed that the presence of HBV resulted in reduced survival, younger median age at death, and a more advanced tumor stage compared to non-HBV etiologies.[9] It was suggested that the biological aggressiveness of HBV-related HCCs might be greater, but in the early stages, curative treatments restrain the difference in tumor progression between HBV and HCV HCC patients.[20]

All existing staging/prognosis systems, including this K-MESIAH, are based on pre-treatment initial evaluations and are limited in their performance after treatment. Treatment could significantly impact survival outcomes, especially palliative treatment of advanced HCC.[21] Fortunately, risk factors for predicting survival outcomes using the MESIAH were developed using a Western cohort collected over 15 years, which means treatment strategies underwent changes, and were validated in a relatively old Korean cohort. The K-MESIAH added etiology to the original risk factors in the MESIAH, which showed a better prediction ability in both the present cohort and another Korean cohort. Therefore, the prediction ability of the K-MESIAH may not be as impacted by treatment. Further prospective evaluation of the K-MESIAH is necessary to determine if treatment does indeed impact it.

In conclusion, the Korean version of the MESIAH performs well in estimating survival in both the development cohort and the validation cohort, where HBV is the main etiology of HCC. This K-MESIAH may be more useful in clinical practice and personalized care of HCC patients in an HBV-endemic area.
